# On Modern Progress in Ophthalmic Medicine and Surgery

**Published:** 1895-01-19

**Authors:** Robert Brudenell Carter

**Affiliations:** Consulting Ophthalmic Surgeon to St. George's Hospital


### ON MODERN PROGRESS IN OPHTHALMIC MEDICINE AND SURGERY.

By ROBERT BRUDENELL CARTER, F.R.C.S., Consulting Ophthalmic Surgeon to St. George's Hospital.

IRITIS—(*concluded from page 183*).

NOTWITHSTANDING all that can be done for the cure of acute iritis, it will sometimes happen that one or more adhesions of the pupillary margin will be left by the first attack, and these, in the majority of instances, are apt to become sources of future trouble. They are most common, of course, when time has been lost prior to the commencement of treatment; and they are most dangerous, according to my experience, when they are few in number and small in size. In the ordinary business of life the diameter of the pupil varies from moment to moment, in accordance with all variations in the amount or brightness of the light which it receives; and a single small adhesion, by constantly checking movements which it is powerless to prevent, is apt to keep the eye in a state of perpetual irritability, and thus to be a predisposing cause of fresh iritis, rendering it liable to occur whenever any exciting cause is brought into operation. The exciting causes, in such circumstances, may be various; and may be such as overwork of the eye, or exposure to cold, or indiscretions of diet of a kind calculated to rouse a rheumatic tendency into activity. The presence of an old adhesion places an insurmountable obstacle in the way of the attainment of the condition most essential to the cure of the iritis, for it prevents the pupil from becoming fully dilated. In the immediate neighbourhood of the adhesion the iris must remain in contact with the capsule of the lens, and here an extension of the original adhesion can scarcely be prevented from occurring. A succession of recurrences may at last tie down the whole of the pupillary margin, and may thus entirely arrest the passage of fluid from the deeper to the more superficial portions of the eyeball. The secretion of the ciliary body accumulates behind the lens and iris, and the retina becomes disorganised and perishes from the pressure to which it is subjected, and from the consequent arrest of its circulation. The state is one of secondary glaucoma, by which sight must almost inevitably be destroyed.

The degree in which an eye is sensitive to the presence of an adhesion varies considerably in different people; and I am of opinion that when a first attack leaves adhesions which are numerous and extensive, they are less worrying than a single one. They probably forbid pupillary movements instead of only impeding them. I have seen a few instances in which even a single adhesion appeared to be innocuous, and certainly still more in which extensive adhesions were not followed by fresh attacks; but no immunity is conferred by adhesions, however extensive, which have themselves been brought about by recurrences of inflammation. The habit of recurrence, when once established, is seldom broken by anything short of an iridectomy, and not always by that. An iridectomy, however, in an eye liable to recurrent iritis, will often break the habit; and when it fails to do so will nevertheless afford great protection against the destructive effects of the subsequent attacks. If a large piece of iris, including its periphery, be removed by operation, it is scarcely possible for the coloboma to be closed by inflammation, and free passage from the posterior to the anterior chamber is almost certain to be maintained.

The subsidence of a first attack of iritis will usually be followed by complete restoration of vision, whether an adhesion be left or not, unless, indeed, the whole of the area of the pupil should be occupied by a film of lymph. When sight is thus restored, the mere presence of an adhesion does not justify an operation, because the adhesion may prove to be harmless. A second attack in the same eye should always, I think, be held to justify, or even to require, the performance of an iridectomy, which, at such a period, can almost always be performed effectually. If many recurrences are permitted to occur, the consequence will be that the iris tissue becomes weak and rotten, so that it will be liable to tear under the forceps, while the adherent portion is likely to be no longer merely marginal, but to be a zone of considerable width. In such conditions the operation has been deferred too long, and it will generally fail to save the eye. The surgeon must consider, of course, the general condition of the patient, the length of interval separating the first and second attacks, and the probable exciting cause of the latter; and considerations arising out of these grounds may render it desirable in certain cases to wait longer than would be prudent in others. But I think there are few exceptions to the rule that an iridectomy is more successful as a precaution against the effects of recurrence the sooner it is accomplished; and my practice for many years has been to advise its performance, in the great majority of cases, as soon as I was convinced that recurrence was to be expected. In patients about to pass away from the vicinity of a skilled operator, as in the case of officers proceeding to remote parts of India, I have been accustomed to recommend operation merely because an adhesion was present, and without waiting for recurrence. In such cases I have never had to regret operation, and I have seen instances in which an operation has been declined, and in which the eyes were subsequently lost.

A common history, in recurrent cases, is that the iridectomy will be followed by a few attacks, generally of constantly diminishing severity, and separated from each other by intervals of increasing length, which are easily amenable to treatment. I have met with instances, however, in which, although a large coloboma has prevented closure of the pupil, the recurrences have been frequent and severe, disqualifying the patient from following his occupation, sometimes for considerable periods of time, and occasioning him much distress and anxiety. In such cases there has mostly been a pronounced rheumatic diathesis, and the most successful treatment has been by the adoption of a mode of life based upon this consideration. In one very severe and obstinate example of this kind a completely vegetable diet was productive of great benefit, and in others the careful protection of the surface against cold, the very moderate consumption of nitrogenous food, limitation of alcohol and complete avoidance of some of its forms, chiefly of malt liquor, together with frequent recourse to a mild mercurial purgative, has succeeded in maintaining a condition of health by which the enemy has for long periods been kept at bay. In all such cases, however, the affected eye has been the weak part of the system, and that in which the consequences of indiscretion were first experienced.

